# Current Insights into Long Non-Coding RNAs in Renal Cell Carcinoma

**DOI:** 10.3390/ijms17040573

**Published:** 2016-04-15

**Authors:** Maximilian Seles, Georg C. Hutterer, Tobias Kiesslich, Karl Pummer, Ioana Berindan-Neagoe, Samantha Perakis, Daniela Schwarzenbacher, Michael Stotz, Armin Gerger, Martin Pichler

**Affiliations:** 1 Department of Urology, Medical University of Graz, A-8036 Graz, Austria; maximilian.seles@medunigraz.at (M.S.); karl.pummer@medunigraz.at (K.P.); 2 Department of Internal Medicine I, Salzburger Landeskliniken (SALK), Paracelsus Medical University, A-5020 Salzburg, Austria; t.kiesslich@salk.at; 3 Laboratory for Tumour Biology and Experimental Therapies, Institute of Physiology and Pathophysiology, Paracelsus Medical University, A-5020 Salzburg, Austria; 4 Department of Experimental Therapeutics, The University of Texas MD Anderson Cancer Center, Houston, TX 77054, USA; ioananeagoe29@gmail.com (I.B.-N.); martin.pichler@medunigraz.at (M.P.); 5 Research Center of Functional Genomics, Biomedicine and Translational Medicine, Iuliu Hatieganu University of Medicine and Pharmacy, 400337 Cluj-Napoca, Romania; 6 Department of Experimental Pathology, The Oncology Institute Ion Chiricuta, 400015 Cluj-Napoca, Romania; 7 Institute of Human Genetics, Medical University of Graz, A-8036 Graz, Austria; samantha.perakis@medunigraz.at; 8 Division of Oncology, Department of Internal Medicine, Medical University of Graz, A-8036 Graz, Austria; daniela.schwarzenbacher@medunigraz.at (D.S.); michael.stotz@medunigraz.at (M.S.); armin.gerger@medunigraz.at (A.G.); 9 Center for Biomarker Research in Medicine, Medical University of Graz, A-8036 Graz, Austria

**Keywords:** renal cell cancer-carcinoma, long non-coding RNAs, metastases

## Abstract

Renal cell carcinoma (RCC) represents a deadly disease with rising mortality despite intensive therapeutic efforts. It comprises several subtypes in terms of distinct histopathological features and different clinical presentations. Long non-coding RNAs (lncRNAs) are non-protein-coding transcripts in the genome which vary in expression levels and length and perform diverse functions. They are involved in the inititation, evolution and progression of primary cancer, as well as in the development and spread of metastases. Recently, several lncRNAs were described in RCC. This review emphasises the rising importance of lncRNAs in RCC. Moreover, it provides an outlook on their therapeutic potential in the future.

## 1. Renal Cell Carcinoma

Renal Cell Carcinoma (RCC) represents the third most common urological malignancy with a worldwide incidence rate of approximately 337,000 cases in 2012. In that particular year, RCC was responsible for an estimated 52,000 deaths in Europe and 145,000 deaths worldwide [1,2]. Despite an observable trend towards early stage diagnosis (“stage migration”), RCC mortality rates have steadily increased over recent decades and have stabilized over the last 10 years. A strong increase in mortality rates has especially been reported for elderly persons aged 60 years and above [3,4,5].

Different histopathological RCC subtypes with distinct genetic driver lesions exist [6], whereby only three subtypes account for approximately 90% of all renal malignancies: clear cell (cc), papillary (p) and chromophobe (ch) RCC.

Clear cell RCC represents by far the most common histopathological subtype which is assumed to arise from the proximal tubule of the nephron [7]. It accounts for ~75% of all solid renal cancers and is a subtype with great intra- and inter-tumoural heterogeneity [8,9,10]. It is frequently associated with loss of chromosome 3p and modifications of the von-Hippel-Lindau (VHL) gene complex, which stabilises the hypoxic-inducible factor (HIF) and thus controls oxygen sensing [10]. Other less frequent cytogenetic alterations are losses of chromosomes 8p, 9p and 14q, as well as gains of chromosomes 5q and 12q [11]. Further genetic alterations include modifications of chromatin-remodelling complexes, such as protein polybromo 1 (PBRM1), BReast CAncer 1 (BRCA1) associated protein-1 (BAP1) and SET (histone methyltransferase specific for lysine-36 of histone H3) domain containing 2 (SETD2) [12].

Papillary RCC (pRCC) Type I and II also arise from the proximal tubule of the nephron and represent approximately 10% of all cases [7,11]. Generally, pRCC type I and II are distinguished by their histopathological features and prognosis [13,14]. Type II tumours can be further classified into three subgroups based on molecular as well as phenotypic characteristics [15]. Additionally, type II tumours tend to present at a more advanced stage and grade with a higher risk of primary metastatic disease and with lower five-year cancer-specific survival (CSS) rates [16,17]. Both entities include inherited forms, such as hereditary pRCC and hereditary leiomyomatosis RCC (HLRCC), in which the met proto-oncogene (MET) and fumarate hydratase are altered [11,18,19]. Type I is associated with MET alterations, whereas Type II tumours are characterized by cyclin-dependent kinase Inhibitor 2A (*CDKN2A*) silencing, *SETD2* mutations, transcription factor E3 (*TFE3*) fusions and increased expression of the nuclear factor-like 2 (NRF2)–antioxidant response element pathway [15].

Chromophobe RCC (chRCC) accounts for ~5% of all renal malignancies and originates from the distal tubule of the nephron [7]. A large-scale genetic investigation found tumour protein p53 (TP53) and phosphatase and tensin homolog (PTEN) to be modified in a relevant number of chRCC patients [20]. This subtype is linked to the Birt-Hogg-Dubé-Syndrome, which is caused by mutations in the folliculin gene, whereas sporadic forms exhibit multiple copy number alterations in chromosomes 1, 2, 6, 10, 13 and 17 [21,22].

In general, treatment options include surveillance, ablative therapies, and, for cases in which there exists a possible and reasonable approach to cure or control disease, surgical partial and radical nephrectomy with or without lymphadenectomy can be performed. [23].

Up to one third of all RCC patients present with primary metastatic disease [24], whereby conventional systemic chemo- and/or radiotherapy shows very moderate to no benefit at all [23]. Nowadays, systemic therapeutic options mainly in ccRCC include targeted therapies, monclonal antibodies against vascular endothelial growth factor (VEGF) and inhibitors for mammalian target of rapamycin (mTOR) [23]. Recently developed agents, such as the small tyrosine kinase inhibitor Cabozantinib and the monoclonal antibody against “programmed cell death protein 1” (PD1) Nivolumab are now ready for daily use [25,26]. Targeted therapies in sequence with mTOR inhibitors and the new agents have demonstrated the best improvements in overall survival (OS). They function via inhibition of single or multiple tyrosine kinases, including e.g., c-kit, platelet derived growth factor (PDGF) and VEGF-Receptors 1, 2 and 3 [25,26,27,28,29]. No reliable molecular prognostic marker has yet been established for RCC [6,23]. Thus, since long non-coding RNAs (lncRNAs) play an emerging role in RCC pathophysiology, their biology in normal human cells, cancer cells and, particularly, their role in RCC shall be discussed.

## 2. Long Non-Coding RNAs (lncRNAs)

Only about 1%–2% of the human genome is protein-coding, while more than 90% is thought to carry non-protein-coding information with other functions or at least with indices of functions [30]. Amongst other reasons, a large portion of the human genome is considered to be potentially non-coding due to missing sequence homologies to known proteins, the absence of open reading frames, and frequent codon substitutions [31]. In order to classify RNAs transcribed from these regions, they are arbitrarily classified into either short or lncRNAs at a length of ±200 nucleotides [32]. The class of short non-coding RNAs comprises microRNAs, which are regarded as small yet highly influential molecules, as previously comprehensively discussed [33,34].

Long non-coding RNAs form a heterogenous group of molecules which influences the gene expression of protein-coding genes in several ways. The genomic locations of lncRNAs can be described according to their relationship to nearby protein-coding genes as intergenic, intronic, bidirectional and sense, as well as antisense [32]. Moreover, lncRNAs exhibit low sequence conservation across different organisms with typically low expression levels, but their function may not uniquely depend on sequence conservation. Their function is location-specific [35], but their mode of action can occur in cis (at their locus) or in trans (at any other locus) within the genome [36]. Regulatory mechanisms include chromatin remodeling (by e.g., XIST (RNA gene on the X chromosome), HOTAIR (HOX antisense intergenic RNA)), transcriptional coactivation, as well as repression (by e.g., H19), protein inhibition (by e.g., TERRA (telomeric repeat-containing RNA)), posttranscriptional modifications (by e.g., MALAT1 (metastasis-associated lung adenocarcinoma transcript 1)) and decoy functions (by e.g., PTENP1 (phosphatase and tensin homolog pseudogene 1)) [37].

At present, a large number of lncRNAs is known to be involved in the initiation, evolvement and progression of cancer, as well as the development and spread of metastases [38]. They seem to control cancer at different cellular and transcriptional levels and they can act as tumour supressors or oncogenes [39]. Many lncRNAs are expressed in a tissue- and cancer-type specific manner and have already been shown to be useful as potential prognostic markers [40,41,42].

### 2.1. Long Non-Coding RNAs in Renal Cell Carcinoma (RCC)

Following the initial identification and characterization of lncRNAs under varying physiological and pathological conditions, several recent studies have sought to find yet undiscovered lncRNAs in RCC tissue. Initially, microarray and then mostly quantitative PCR methods were used to identify a large number of novel lncRNAs in RCC tissue [43,44,45,46]. Up to 35,000 different lncRNAs were found in each study, but only some hundreds up to thousands were found to be differentially expressed compared to normal renal tissue samples. The number of down- as well as up-regulated lncRNAs in each patient and publication has shown great variation, for which no general conclusion for common expression patterns can be drawn [43,44,45]. Neither comparison to clinical parameters nor knock-down of the most abundant lncRNAs involved in cell migration has yet provided further information for finding common promising lncRNAs, which might be attributed to the high variability in terms of RCC biology, as discussed above [43].

#### 2.1.1. A Novel lncRNA-Based Subclassification of Clear-Cell RCC (ccRCC) [47]

Very recently, ccRCC was categorised into four distinct subgroups based on their lncRNA expression patterns: C1 (29.3%), C2 (23.4%), C3 (39.6%) and C4 (7.8%) [47]. Computational analysis revealed gene enrichment associated with the G2/M cell cycle phase for the C2 subgroup and genes associated with pathways relevant for the development of the early distal tubule of the nephron for C4. In particular, the C4 subtype has different cytogenetic alterations when compared to C1, C2 and C3: C4 contains a mixture of microphthalmia-associated transcription factor (MITF/TFE) translocation RCC, chRCC and clear cell papillary RCC, indicating a previous incorrect pathological diagnosis of ccRCC [47]. According to the authors, their lncRNA-based classification correlated well with the TCGA (The Cancer Genome Atlas) transcriptome classification of ccRCC. This classification was created with data obtained from 500 RCC samples which underwent a comprehensive molecular characterization [12]. When correlated with clinical data, C2 represented the most aggressive subtype by far with statistically significant, higher Fuhrman grade, pathological size, higher rate of lymph node involvement and metastasis, higher tumour, node, metastasis classification system (TNM) stage and worse OS.

#### 2.1.2. Specific lncRNAs in RCC

In the following section, we will discuss lncRNAs which have already been described in detail in RCC with regard to their special function, as well as their possible clinical use. Table 1 shows an up-to-date list of all lncRNAs including their main location and main function.

##### HOX Antisense Intergenic RNA (HOTAIR)

“HOX antisense intergenic RNA” (HOTAIR) is transcribed from the antisense strand of the HoxC gene located on chromosome 12 and was the first lncRNA whose role in cancer was discovered [48]. HOTAIR interacts with enhancer of zeste homolog 2 (EZH2), a subunit of the polycomb repressive complex 2 (PRC2), which leads to histone 3 lysine 27 (H3K27) trimethylation of the HOXD locus, causing transcriptional repression [49,50]. HOTAIR has been reported to be overexpressed in various types of cancer and represents a promising potential prognostic marker [48,51,52,53].

Interestingly, HOTAIR expression is elevated in RCC cells compared to normal renal tissue [54]. HOTAIR knock-down by siRNA demonstrated reduced migration and decreased proliferation with cell cycle arrest at the G0/G1 phase and with significantly less cells in the G2/M phase. HOTAIR overexpression results in higher concentrations of p53, p21 and p16 mRNA and lower concentration of EZH2 with lower binding strength in RCC cells *in vitro*. Renal cell carcinoma tumour xenografts with stimulated HOTAIR expression which were previously injected into mice were smaller and exhibited reduced proliferation [54]. Curcumin inhibited HOTAIR-induced cell migration in a dose-dependent manner without any signs of toxicity *in vivo* [55].

HOTAIR represents the archetype of all lncRNAs and has already been used in tumour transplantation models [54].

##### Metastasis-Associated Lung Adenocarcinoma Transcript 1 (MALAT1)

The “metastasis-associated lung adenocarcinoma transcript 1” (MALAT1), also known as “nuclear-enriched abundant transcript 2” (NEAT2), represents a ubiquitously expressed lncRNA, showing high conservation across several species [56]. Recently, intensive research has focused on MALAT1, and it has been linked to many types of human cancers [56,57,58,59]. In general, the expression of MALAT1 is higher in cancerous tissue compared to normal tissue with enhancement of cell proliferation, apoptosis, migration, invasion and metastatic spread of tumour cells. Furthermore, it might be used as a prognostic clinical parameter [56,57,59,60,61]. In accordance with other cancer types, MALAT1 is also up-regulated in ccRCC samples and in RCC cell lines compared to non-tumourous renal tissue or cells [62,63,64]. A knock-down of MALAT1 in RCC cell lines inhibits cell proliferation, migration and invasion; moreover, it increases apoptosis rates [62,63,64]. Hirata *et al.* 2015 were able to show a direct activation of MALAT1 by c-fos, a transcription factor activated in conjunction with c-jun in the downstream pathway of VHL tumour suppressor gene inactivation in ccRCC [63,65]. Together, they drive Twist protein expression and thus induce epithelial-to-mesenchymal transition (EMT) [65].

**Figure 1:**
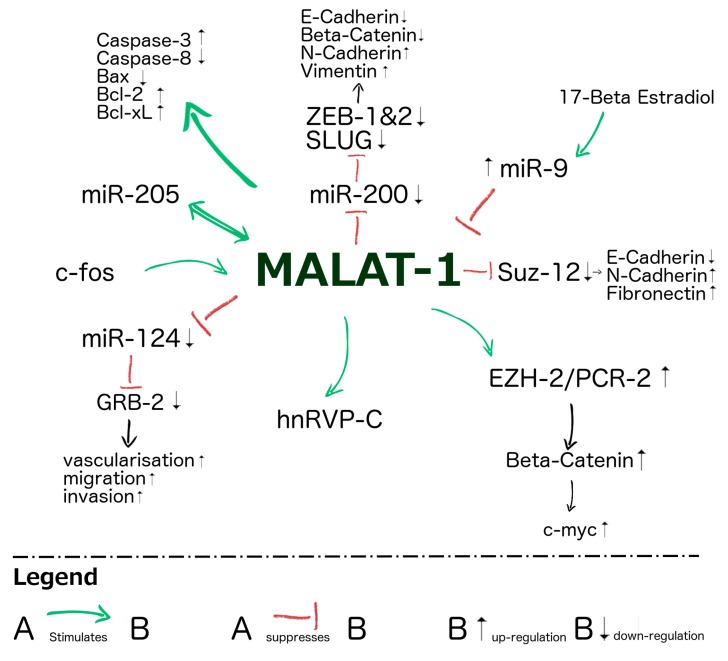
Schematic drawing showing different pathways with involvement of long non-coding RNA (lncRNA) metastasis-associated lung adenocarcinoma transcript 1 (MALAT1). Green arrows indicate stimulation, red “T” indicates suppression, black vertical arrows indicate up- or down-regulation [57,63,64,72,73,74,75,76,77].

Other lncRNAs, such as H19 and HOTAIR, are known to bind to PRC2 to induce gene silencing via H3K27 trimethylation [50,66,67]. PRC2 has several subunits and MALAT1 has been shown to bind to one of them in bladder cancer [67,68]. Recently, a direct correlation between MALAT1 and EZH2, another subunit of PRC2, was shown. After inactivation of MALAT1, decreased expression of EZH2, beta-catenin, H3K27me3 and c-myc in contrast to increased levels of E-cadherin could be demonstrated [69]. E-cadherin represents a tumour suppressor gene which is typically down-regulated during EMT in RCC [69]. β-Catenin is part of the Wnt/β-catenin pathway which is activated by EZH2 through accumulation of dephosphorylated beta-catenin to bind T-cell factor/lymphoid enhancer factor transcription factors (TCF/LEF). Consequently, this activates Wnt target genes such as c-myc to drive carcinogenesis [70,71]. Furthermore, a reciprocal effect between MALAT1 and miRNA-205, a tumour suppressor in RCC, was observed. Acting as a competing endogenous RNA (ceRNA), MALAT1 regulates zinc finger E-box-binding homeobox 2 (ZEB2) expression via sponging of miR-200 in a dose-dependent manner and thus has a significant function in EMT in RCC [64]. All these results show a possible means as to how MALAT1 induces carcinogenesis by facilitating EMT, cancer progression and metastasis. Figure 1 shows different pathways regarding MALAT1’s involvement at the cellular level.

Interestingly, a fusion of the Alpha Gene/MALAT1 with the basic helix-loop-helix leucine zipper (bHLH-LZ) transcription factor EB (TFEB) based on chromosomal translocation of t(6; 11)(p21; q13) was observed in pRCC as well as in paediatric RCC [78,79]. In addition to the mentioned molecular insights, high levels of MALAT1 are significantly correlated with tumour size, pathologic T-stage, as well as lymph node metastases [62]. In a multivariate analysis, high MALAT1 expression represented an independent prognostic factor for shorter OS [62,63].

MALAT1 is one of the few well-described lncRNAs that could be considered for serious further investigation in terms of finding a reliable molecular marker and a promising therapy in RCC. This has already been demonstrated in lung cancer, where antisense oligonucleotide blocking of MALAT1 prevented the spread of metastasis after tumour implantation in a mouse xenograft [80].

##### H19

H19 represents a maternally imprinted lncRNA from the Igf2/H19 imprinted gene cluster at the telomeric end of chromosome 11. It is expressed during the embryonic period, but is then completely repressed in most human tissues after birth [81,82,83]. Hypoxic-inducible factor 1 alpha (HIF1A) acts as a major trigger for H19 expression under hypoxic stress. When combined with mutation of p53, HIF1A promotes angiogenesis, tumourigenesis, multidrug resistance and the ability of cells to metastasise and survive. miR-675 is a microRNA expressed from the same gene locus as H19 and suppresses the tumour suppressor protein retinoblastoma (Rb) [84]. H19 also plays a key role in mesenchymal-to-epithelial transition (MET) as well as EMT by influencing the function of EZH2, β-catenin and E-cadherin [81]. H19 is up-regulated in several types of cancer, including breast, bladder, ovarian and gastric cancer, as well as glioma. H19 has been a target of promising vector-based genetic therapy carrying the gene for diphtheria toxin A. It has been successfully used in phase I and II trials in bladder, pancreatic and ovarian cancer, as well as in metastatic colon cancer [85,86,87,88].

In RCC, H19 is upregulated compared to adjacent human renal tissue and normal renal cell line tissue. Knock-down of H19 results in decreased growth, migration, invasion, and in reduced wound healing capacity. When linked to clinical data, a significant correlation in terms of tumour stage and the presence of lymph node and distant metastases could be shown. Higher expression of H19 is associated with significantly shorter OS [89].

In contrast to other lncRNAs, H19 has already been found to be a promising target for gene therapy in various types of cancer. Targeting cancer by vector-based gene therapy with the help of H19 could also represent a potential approach for targeting RCC in the future.

##### Hypoxic-Inducible Factor 1 Alpha (HIF1A) Transcripts

HIF is a dimeric transcription factor and mainly regulates the cellular response to low oxygen concentrations. Its subunit HIF1A is the main oxygen sensor and is up-regulated in various tumours [90]. Inhibition of topoisomerase II after induced DNA damage yields elevated levels of two new lncRNAs at the 3′ and 5′ ends antisense to HIF1A, named 5′ HIF1A and 3′ HIF1A [91,92]. 5′ HIF1A accumulates at the perinuclear cellular compartment and co-localises with the nuclear pore complex Nup62 protein, suggesting a role in nuclear membrane trafficking. 5′ HIF1A and 3′ HIF1A are expressed at high and intermediate levels in non-papillary RCC, respectively, and 3′ HIF1A is expressed at low levels in pRCC [91,92]. Information regarding a correlation to clinical data is not yet available. More precise insight into the function of all HIF1A transcripts in RCC is warranted. With this information, a correlation to patient data can be made and this, in turn, will possibly poin to the prospective role of these transcripts in the future.

##### Growth Arrest Specific 5 (GAS5)

The “Growth Arrest Specific 5” (GAS5) gene encodes for both short non-coding RNAs as well as for lncRNAs and is alternatively spliced into two smaller molecules , namely GAS5a and GAS5b [93,94]. It is located on chromosome 1 and is responsible for stimulation of apoptosis via p53 and Baculoviral IAP repeat-containing protein 3 (cIAP2), as well as for inhibition of cell proliferation via p21, CDK6 and cyclin D1 [93,95]. It functions as a decoy to inhibit steroid receptor-induced activity or activity of miR-21 and serves as a regulator of translation via Eukaryotic translation initiation factor 4E (eIF4E) and c-myc [94]. Recent investigations have even suggested a coding function for micropeptides, which makes its non-coding nature questionable [96].

The function of GAS5 as a tumour suppressor has also been confirmed in RCC. GAS5 expression levels are significantly lower in ccRCC samples *in vitro* and *in vivo* when compared to non-tumorous renal tissue [97]. After cell stimulation with a plasmid-controlled DNA vector *in vitro*, the well-known growth-suppressing effect on cell proliferation, apoptosis and cell cycle duration was demonstrated [97]. However, a link to real clinical data which would provide further possibilities for predicting outcomes in RCC patients could not be demonstrated [97].

GAS5 represents an interesting gene with many functions yet to be discovered. However, it is doubtful whether it will play a significant role in RCC, since significant correlations with clinical data have not yet been made.

##### Maternally Expressed Gene 3 (MEG3)

“Maternally expressed gene 3” (MEG3) represents a ubiquitously expressed lncRNA that is known to influence many functions of human embryonic, stem cell and mature human tissue in several physiological conditions, including neurogenesis and insulin synthesis [98,99,100]. It is located on chromosome 14 and is known to act as a tumour suppressor through activation of p53 in several human cancers such as e.g., lung cancer and glioma [101,102,103].

In RCC, MEG3 is significantly down-regulated in comparison to normal renal tissue *in vivo* and in cultured cells [104]. *In vitro*, the apoptosis rate is significantly increased after transfection of MEG3 into cells [104]. This higher apoptosis rate could be explained by the activation of the intrinsic mitochondrial pathway, shown by reduced expression of B-cell lymphoma 2 (BCL2) and procaspase-9 proteins as well as enhanced expression and release of caspase-9 protein and cytochrome-c into the cytoplasm [104]. No data has yet been correlated to actual patient characteristics and, for this reason, clinical significance still remains to be elucidated.

In the future, it will first be necessary to link the abovementioned results regarding MEG3 at the cellular level to real-life patient data in order to identify if any significant correlations exist. If this is the case, further investigation is needed to clarify the future role of MEG3 in RCC.

##### Protein Sprouty Homolog 4 Intronic Transcript 1 (SPRY4-IT1)

“Protein sprouty homolog 4 intronic transcript 1” (SPRY4-IT1), which is transcribed from the second intron of the SPRY4 gene at chromosome 5, is an inhibitor of the receptor-transduced mitogen-activated protein kinase (MAPK) signalling pathway [105]. It is up-regulated in several types of cancer and knock-down of its expression leads to cell growth arrest, inhibition of invasion and elevated rates of apoptosis [106,107]. It plays a pivotal role in EMT by regulating E-cadherin and vimentin expression in glioma [108].

In RCC, SPRY4-IT1 is found in higher levels in ccRCC tissue and in RCC cell lines [109]. Knock-down of SPRY4-IT1 results in reduced cell migration, proliferation and invasion. High expression of SPRY4-IT1 in ccRCC correlates significantly with histological grade, tumour stage, the presence of infiltrated lymph nodes, distant metastases, as well as OS [109].

Given that there is only one single publication regarding SPRY4-IT1 in RCC, the potential role of this non-coding RNA remains difficult to define. Further investigation is necessary before any conclusion can be drawn.

##### Cell Adhesion Molecule 1 Antisense Transcript 1 (CADM1-AS1)

Long non-coding RNA “Cell adhesion molecule 1 antisense transcript 1” (CADM1-AS1) is located in the antisense direction of the exon coding for CADM1, which resides on chromosome 11. CADM1 represents a membrane protein involved in cell-to-cell interactions and it is known for its function as a tumour suppressor in several types of cancer [110].

In ccRCC specimen, CADM-AS1 levels are lower than in adjacent normal renal tissue, while CADM mRNA levels even differ to a significant extent. Transfection with CADM1-AS1 siRNA resulted in significantly lower expression of CADM1-AS1 lncRNA and CADM1 mRNA. Transfection with pcDNA (DNA vector used to clone recombinant DNA sequences) CADM1-AS1 resulted in lower migration and apoptosis rates as well as lower growth rates. Lower CADM1-AS1 levels are significantly correlated with OS, clinical stage and tumour diameter [111].

The same issue surrounding SPRY4-IT1 also applies to CADM-AS1. With only one single manuscript published, it is not feasible to accurately predict its further role in diagnostics and therapy. Thus, a validation of the above findings along with an improved molecular characterisation of CADM-AS1 is strongly warranted.

##### Renal Cell Carcinoma Related Transcript 1 (RCCRT1)

The lncRNA “Renal cell carcinoma related transcript 1” (RCCRT1), whose exact function is currently unknown, is located on chromosome 5. It shows significantly higher expression in ccRCC tissue compared to normal adjacent renal tissue. Furthermore, knock-down of its expression results in lower cell migration and invasion *in vitro*. Significant correlation between clinical characteristics and RCCRT1 expression could be found in terms of pathologic T-stage as well as lymph node and distant metastases [112].

In the case of RCCRT1, very little is known about the lncRNA itself and information regarding its function. A basic characterisation is necessary before any conclusion for its future role in RCC can be drawn.

##### Neuroblastoma Associated Transcript 1 (NBAT1)

The lncRNA “Neuroblastoma associated transcript 1” (NBAT1) is located on chromosome 6 and is known to control neuroblastoma progression by regulating cell proliferation and neuronal differentiation [113].

In RCC, NBAT was found at a significantly lower expression level in cancerous tissue than in normal adjacent tissue. Knock-down of NBAT by siRNAs results in increased cell proliferation, migration and invasion *in vitro*. Significantly lower NBAT1 levels were found to be correlated with histological grade, tumour stage, lymph node metastases and were also associated with significantly worse OS [114].

Due to its significant correlation with real patient data, NBAT1 might be a promising candidate for further research as a biomarker or therapeutic agent.

## 3. Conclusions

Beyond doubt, lncRNAs have an important role in different types of cancer, including RCC, with regard to the underlying biology, cancer initiation and progression to distant metastases. Despite all hopes and recent developments in lncRNA research, the functional role of lncRNAs remains unclear. As described above, lncRNAs can be linked to various physiological and pathological functions. However, in the end, phenotypic expression and its consequence for the individual is of paramount importance. In order to investigate phenotypic expression, lncRNAs have to be manipulated to examine their potential implications. This can be achieved using various approaches, e.g., deletion of the promoter region or the whole gene, integration of a premature polyadenylation sequence, antisense oligonucleotide blocking, and others [80,115].

Several examples for lncRNA knockout cultured cell and animal models with and without phenotypic changes are now available. For example, Neat1, a very abundant lncRNA in close relation to MALAT1, is required for the development of the mammary glands and corpus luteum, as well as for the possibility of lactation and the establishment of pregnancy in mice [116,117]. On the other hand, knockout of MALAT1 does not result in obvious alterations of pre- and post-natal development in mice [118,119,120,121]. Knockout of HOTAIR results in viable mice but with transformation of the spinal vertebrae and metacarpal bones, while knockout of Fendrr (Foxf1 adjacent non-coding developmental regulatory RNA) results in embryonic lethality [122,123].

As only a minor fraction of lncRNAs have been investigated thus far, a final conclusion explaining in detail lncRNA functions and their role in physiological and pathological processes is not yet possible.

Ultimately, all efforts are carried out with the goal of improving cancer management in humans. To date, not a single lncRNA has made its way into at least urological guideline-based clinical routine practice so far [23,124,125,126], but for different types of cancer, a few very promising candidates do exist [127,128,129]. Additionally, various approaches have been pursued to potentially use lncRNAs as therapeutic agents in different types of cancer. These approaches include e.g., small interfering RNAs, ribozymes, aptamers, antisense oligonucleotides, natural antisense transcripts and small molecules [127,129]. Equally, these agents have not yet found their way into routine clinical oncological use.

In 2016, lncRNAs in RCC still remain in their infancy with a few promising candidates offering potential application as biomarkers or novel therapeutic targets. A number of basic as well as applied research studies still need to be performed in order to fully understand the underlying mechanisms of their functions before clinical use of lncRNAs in RCC patients becomes a reality.

## Figures and Tables

**Table 1:** Table listing all currently known lncRNAs in RCC showing their main location and main function.

Name	Location	Tumour Suppressor/Oncogene	Function
HOTAIR	12q13.13	Oncogene	PRC 2 control
MALAT1	11q13.1	Oncogene	PRC 2 control
H19	11p15.5	Oncogene	Embryonic growth factor
GAS5	1q25.1	Tumour suppressor	Stimulation of apoptosis, inhibition of cell proliferation
MEG3	14q32	Tumour suppressor	Stimulation of apoptosis
SPRY4-IT1	5q31.3	Oncogene	Inhibition of MAPK pathway
CADM1-AS1	11q23	Tumour suppressor	Cell to cell interaction
RCCRT1	chr5: 137801181–137805004	Oncogene	not known
NBAT1	6p22	Tumour suppressor	Control of cell proliferation & neuronal differentiation

## Abbreviations

RCC	renal cell carcinoma
ccRCC	clear-cell RCC
pRCC	papillary RCC
chRCC	chromophobe RCC
VHL	von-Hippel-Lindau
HIF	hypoxic-inducible factor
PBRM1	protein polybromo 1
BAP1	BRCA1 associated protein-1
SETD2	SET domain containing 2
CSS	cancer-specific survival
HLRCC	hereditary papillary RCC and as hereditary leiomyomatosis and RCC
MET	met proto-oncogene
CDKN2A	cyclin-dependent kinase Inhibitor 2A
TFE3	transcription factor E3
NRF2	nuclear factor-like 2
TP53	tumour protein p53
PTEN	phosphatase and tensin homolog
VEGF	vascular endothelial growth factor
mTOR	mammalian target of rapamycin
PD1	programmed cell death protein 1
OS	overall survival
PDGF	platelet derived growth factor
lncRNAs	long non-coding RNAs
XIST	RNA gene on the X chromosome
HOTAIR	HOX antisense intergenic RNA
H19	H19 gene
TERRA	telomeric repeat-containing RNA
MALAT1	metastasis-associated lung adenocarcinoma transcript 1
PTENP1	phosphatase and tensin homolog pseudogene 1
PCR	polymerase chain reaction
MITF/TFE	microphthalmia-associated transcription factor
TCGA	the Cancer Genome Atlas
TNM	tumour, node, metastasis classification system
EZH2	enhancer of zeste homolog 2
PRC2	polycomb repressive complex 2
siRNA	small interfering RNA
mRNA	messenger RNA
NEAT2	nuclear-enriched abundant transcript 2
EMT	epithelial-to-mesenchymal transition
TCF/LEF	T-cell factor/lymphoid enhancer factor transcription factors
ceRNA	competing endogenous RNA
ZEB2	zinc finger E-box-binding homeobox 2
HIF	hypoxic inducible factor
GAS5	growth arrest specific 5
cIAP2	Baculoviral IAP repeat-containing protein 3
CDK6	cell division protein kinase 6
pcDNA	DNA vector used to clone recombinant DNA sequences
MEG3	maternally expressed gene 3
BCL2	B-cell lymphoma 2
SPRY4-IT1	protein sprouty homolog 4 intronic transcript 1
MAPK	mitogen-activated protein kinase
CADM1-AS1	Cell adhesion molecule 1 anti-sense transcript 1
RCCRT1	renal cell carcinoma related transcript 1
NBAT1	neuroblastoma associated transcript 1
Neat1	nuclear-enriched abundant transcript 1
Fendrr	Foxf1 adjacent non-coding developmental regulatory RNA
